# Genetics of Dothistromin Biosynthesis in the Peanut Pathogen *Passalora arachidicola*

**DOI:** 10.3390/toxins2122738

**Published:** 2010-11-29

**Authors:** Shuguang Zhang, Yanan Guo, Rosie E. Bradshaw

**Affiliations:** Bio-Protection Research Centre, Institute of Molecular BioSciences, Massey University, Palmerston North, New Zealand; Email: s.zhang@irl.cri.nz (S.Z.); Y.Guo.1@massey.ac.nz (Y.G.)

**Keywords:** aflatoxin, gene cluster, gene regulation, mycotoxin, *Dothistroma*

## Abstract

The peanut leaf spot pathogen *Passalora arachidicola* (*Mycosphaerella arachidis*) is known to produce dothistromin, a mycotoxin related to aflatoxin. This is a feature shared with the pine needle pathogen *Dothistroma septosporum* (*Mycosphaerella pini*). Dothistromin biosynthesis in *D. septosporum* commences at an unusually early stage of growth in culture compared to most other fungal secondary metabolites, and the biosynthetic genes are arranged in fragmented groups, in contrast to aflatoxin gene clusters. Dothistromin biosynthetic genes were identified and studied in *P. arachidicola* to determine if the attributes described in *D. septosporum* are shared by another dothistromin-producing species within the Class Dothideomycetes. It was shown that dothistromin biosynthesis is very similar in the two species with regard to gene sequence and gene synteny. Functional complementation of *D. septosporum* mutants with *P. arachidicola* dothistromin genes was also possible. These similarities support a vertical mode of dothistromin gene transmission. *P. arachidicola* also produced dothistromin at an early growth stage in culture, suggesting that this type of regulation pattern may be relevant to the biological role of dothistromin.

## 1. Introduction

Many plant pathogenic fungi produce secondary metabolites such as mycotoxins and phytotoxins. The ability to produce a particular type of metabolite is usually confined to specific taxa, but the distribution of metabolite production within taxonomic groups is often ‘patchy’ [1]. Some secondary metabolites have a role in disease. There are many examples of host-selective toxins (active only against specific susceptible host cultivars or species) that are virulence factors for disease [1,2]. However, in general, the role of non-host-selective toxins is not so clear [3]. Dothistromin is a non-host-selective toxin that is toxic to a broad range of cell types [4]. It is produced by several species with teleomorphs in the genus *Mycosphaerella* including the pine needle pathogens *Dothistroma septosporum* and *Dothistroma pini*, and the peanut leaf spot pathogen *Passalora arachidicola* (previously called *Cercospora arachidicola*; teleomorph *Mycosphaerella arachidis*) [5]. Studies with dothistromin-deficient mutants of *D. septosporum* showed that dothistromin is not required for disease in pine needles [6], despite the prolific production of dothistromin seen in ‘red bands’ of needles with Dothistroma disease [7]. The role of dothistromin in pathogenicity of *P. arachidicola* to its peanut host is not known and to the best of our knowledge no toxin-deficient mutants have been found or transformation systems developed for this pathogen that would allow this to be determined.

One clue that might help discern the biological role of dothistromin is the unusual pattern of regulation seen in *D. septosporum*. Dothistromin is produced mainly during early exponential phase of growth in culture [8]. This is in contrast to its chemical cousin aflatoxin, which is produced primarily during late exponential/stationary phase growth of *A. flavus/parasiticus*, as is typical for secondary metabolites [9]. Laboratory studies suggest a role for dothistromin in competition against other organisms such as needle endophytes and other latent pathogens [6]. Our working hypothesis is that dothistromin is produced during a period of rapid fungal growth that occurs after release of nutrients from plant cells and *in planta* studies are in progress to test this. 

Analysis of the dothistromin genes that have been discovered in *D. septosporum* so far has not revealed any clues to its atypical regulation. Like orthologous aflatoxin (AF) and sterigmatocystin (ST) genes, many of the dothistromin genes have putative binding sites for AflR, a key regulatory protein in AF/ST biosynthesis [10]. However there is a major difference in the arrangement of AF/ST and dothistromin genes. Whilst most of the known AF and ST biosynthetic genes are tightly clustered in *Aspergillus* [11], dothistromin genes are dispersed in several mini-clusters. The mini-clusters are co-located on a 1.3 Mb chromosome and each mini-cluster also contains genes with functions unrelated to dothistromin production [10]. Fragmented metabolite gene clusters have been reported in other fungi, such as lolitrem biosynthesis in *N. lolii* [12]. However, due to the high level of similarity between dothistromin and AF/ST genes, this marked difference in gene organization leads to interesting questions about whether the gene arrangement affects regulation of gene expression, as well as how these gene clusters evolved.

In general, fungal gene clusters are considered to have arisen by vertical transmission from a common ancestor with relocation, recombination, duplication and/or loss of genes and there is evidence for this mode of transmission for the aflatoxin gene cluster [11,13,14]. The currently accepted hypothesis is that AF/ST and dothistromin gene clusters arose by vertical transmission with progressive recruitment of genes to a clustered region [13,15]. However the observation that dothistromin is produced by *P. arachidicola* (Class Dothideomycetes) presents an intriguing link with aflatoxin production by *A. flavus* and *A. parasiticus* (Class Eurotiomycetes) because they can both infect the peanut plant *Arachis hypogaea* [16,17]. Such co-occurrence leads to the potential for horizontal transfer of genes between these disparate Classes of fungi.

The aims of the current investigation were two-fold. The first aim was to determine whether the production of dothistromin at an early stage of growth is an unusual feature confined to *D. septosporum*, or whether *P. arachidicola* shares a similar pattern of regulation. If our hypothesis that dothistromin has a role in competition is correct, we predict that the timing of dothistromin biosynthesis may be important and therefore conserved between related plant pathogens that produce this metabolite. The second aim was to compare dothistromin gene organization between *D. septosporum* and *P. arachidicola*. The hypothesis of vertical transmission of AF/ST/dothistromin clusters leads to the prediction of greater similarities in gene sequence, clustering and synteny between the closely-related *P. arachidicola* and *D. septosporum* than between *P. arachidicola* and the AF-producers *A. parasiticus* and *A. flavus*.

## 2. Materials and Methods

### 2.1. Strains, Culture Conditions and Densitometric Quantification of Dothistromin

*Passalora arachidicola* isolate ATCC18667 was obtained from the American Type Culture Collection (Manassas, Virginia) and maintained at Massey University as strain NZ46. Growth was on potato dextrose agar (PDA) at 22 °C unless otherwise stated. For broth cultures, one 5-mm diameter plug of mycelium was macerated with a pestle in H_2_O and used to inoculate between two and four 125 mL flasks containing 25 mL potato dextrose (PD) broth for genomic DNA preparation or Dothistroma medium (DM; [18]) for monitoring growth and gene expression. Cultures were grown at 22 °C and 160 rpm for 14 days (gDNA preparation) or for up to 30 days with three biological replicates per time point (growth experiment), then filtrates and mycelia harvested by filtration through Miracloth (Calbiochem Corporation, La Jolla, CA). Mycelia for RNA extraction were snap-frozen in liquid nitrogen and those for DNA extraction or biomass quantification were freeze-dried. For quantification, dothistromin was extracted from 5 mL filtrate using three successive treatments with 5 mL chloroform, air-dried and dissolved in 50 µL of DMSO. TLC was carried out as described in [22] with serial dothistromin standards. The densitometric values for each dothistromin band on the TLC plate were determined using a BioRad Gel Doc Documentation (BioRad) and Gel-Pro Analyser (Media Cybernetics, Inc., Bethesda, MD).

### 2.2. Identification of Dothistromin Gene Fragments by Degenerate PCR

Genomic DNA was isolated from NZ46 using a CTAB method [19]. Fragments of putative *P. arachidicola cypA*, *dotA*, *vbsA* and *pksA* genes were PCR amplified using degenerate primers designed from alignments of homologous *A. parasiticus*, *A. nidulans* and *D. septosporum* genes. PCRs were performed in 25 µl reactions containing 0.5 U *Taq* DNA polymerase (Invitrogen, Carlsbad, CA), 1× Invitrogen PCR buffer, 1.5 mM MgCl_2_, 50 mM dNTPs, 0.4 mM of each primer (see Supplementary Table S1) and 10 ng NZ46 genomic DNA. Amplification was done with a gradient Mastercycler (Eppendorf, Hamburg) with an initial step of 94 °C for 2 min, followed by three cycles of 94 °C for 30 s, 66 °C for 60 s, and 72 °C for 80 s. In subsequent cycles the c 3 °C every three cycles down to 42 °C, followed by 72 °C for 5 min. PCR products were purified using a QIAquick PCR Purification Kit (Qiagen, Hilden, Germany), ligated into pGEM-T easy vector (Promega Corp., Madison, WI), transformed into *E. coli* Top10 (Invitrogen), plasmids extracted using a QIAprep spin miniprep kit (Qiagen) and sequenced using an ABI Prism Big-Dye Terminator cycle sequencing ready reaction kit and ABI3730 Genetic Analyzer (Applied Biosystems, Foster City, CA). 

### 2.3. Isolation and Identification of Clustered Dothistromin Genes

Clones containing genes clustered alongside the dothistromin gene fragments identified by degenerate PCR were obtained by construction and screening of size-fractionated genomic libraries. Southern hybridization was firstly carried out to determine the best libraries and fractions to use for each available gene fragment and to assess copy number. *P. arachidicola* NZ46 genomic DNA (2 µg) was digested with EcoRI, SalI, BamHI or ScaI and fractionated on a 0.7% agarose gel. Southern hybridization using DIG-labeled probes (Roche) amplified from dothistromin genes *dotA*, *cypA*, *pksA* and *vbsA* was done as described previously [10]. For partial genomic library construction, 6–10 kb and 10–14 kb EcoRI-digested DNA fractions were recovered using a QIAquick gel extraction kit (Qiagen), ligated into plasmid vector pIC19H and cloned in *E. coli* Top10. Libraries were screened by plate colony hybridization as described [10] using the same DIG-labeled probes as above. Plasmid DNAs were isolated and sequenced on both strands as above, using a primer walking strategy. 

Based on these sequenced plasmids and the degenerate PCR products, inverse PCR was carried out as described by Ochman *et al.* [20] to extend the sequenced regions. Genomic DNA was digested with restriction enzymes EcoRI, HindIII, KpnI and NcoI, respectively, then self-ligated with T4 DNA ligase (Roche) at 4 °C overnight. PCR reactions were carried out as described in Section 2.2 using primers SZDP-82 and SZDP-83 with EcoRI ligation for extension from *Pa-dotA*, primers MGPA22 and MGPA35 with KpnI ligation for *Pa-pksA*, primers MGPA66 and MGPA69 with NcoI ligation for *Pa-moxA* and primers MGPA46 and MGPA79 with HindIII ligation for both *Pa-hexA* and *Pa-vbsA* ends (for primers, see Supplementary Table S1). PCR products were cloned using a pGEM-T easy vector and then sequenced.

### 2.4. RNA Isolation and RT-PCR

Total RNA was isolated from frozen mycelia using TRIzol reagent (Invitrogen) and quantified using a NanoDrop spectrometer (NanoDrop Technologies, Wilmington, Delaware). RNA quality was assessed on a sodium dodecyl sulfate (SDS; 0.3%) tris-acetate-EDTA (TAE) buffer agarose gel before treatment with TURBO DNase (Ambion, Austin, TX). For first strand cDNA synthesis, 500 ng DNase-treated total RNA was reverse transcribed using random hexamer primers and superscript^TM^ III RT (Invitrogen) in a 20 µL reaction, following the manufacturer’s instructions. Semi-quantitative RT-PCR was performed to measure the expression of *Pa-dotA*, *Pa-pksA*, *Pa-vbsA* and control β-tubulin (*Pa-tub*; accession number HM587712) genes using primers listed in Supplementary Table S1. To exclude false positive PCR products from contaminating genomic DNA, at least one primer of each pair for each gene was designed to exon regions flanking an intron. PCR reactions were set up as in Section 2.2 above except with 1 µL of diluted cDNA reaction from each time point as template. The dilution of the individual cDNA samples was determined empirically to show exponential phase amplification for each gene at 30 cycles, and assessed in triplicate. Cycling conditions were 2 min at 94 °C; 30 cycles of 30 s at 94 °C, 30 s at 60 °C, and 60 s at 72 °C; followed by 5 min at 72 °C. PCR samples were electrophoresed on 2.5% agarose TBE gels, stained with ethidium bromide (10 µg/mL) and densitometric values for each PCR product determined as described in Section 2.1. RT-PCR values are presented as a ratio of the dothistromin gene signal relative to that of β-tubulin.

### 2.5. Preparation of NZ46 *Pa-dotA* and *Pa-vbsA* Gene Complementation Constructs

The *Pa-dotA* complementation plasmid pR280 was constructed with a *D. septosporum dotA* promoter region (transcriptional fusion) using a three-step procedure. Primer sequences are shown in Supplementary Table S1. Firstly a region including the open reading frame (904 bp) and 3’ UTR (372 bp) of the *Pa-dotA* gene was PCR amplified using primers SZDP-137 and SZDP-130 and 962 bp of the promoter region of the *Ds-dotA* gene was amplified with primers SZDP-135 and SZDP-136. Primers SZDP-136 and SZDP-137 overlapped both amplicons and were complementary to each other. In the second step, the two PCR products were combined in a further round of PCR using primers SZDP-135 and SZDP-130. Finally, the combined 2.2 kb product was cloned using the pGEM-T easy vector (Promega) to make plasmid pR280, and the sequence confirmed by DNA sequencing. For complementation of *D. septosporum vbsA* KO FJT12, plasmid pR283 containing the 5.8 kb EcoRI fragment (556–6321 bp, GenBank accession No. GU566731), constructed initially for dothistromin gene cloning, was used directly.

### 2.6. Functional Identification of *P. Arachidicola* Dothistromin Genes by Complementation of D. Septosporum Mutants

**Figure S1:**
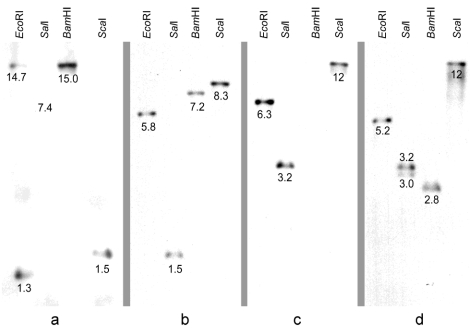
Southern hybridization of digested *P. arachidicola* gDNA with DIG-labeled dothistromin gene fragments *Pa-dotAv* (**a**), *Pa-vbsA* (**b**), *Pa-cypA* (**c**) and *Pa-pksA* (**d**). Each gene appears to be present in only one copy in the genome. On the basis of these results, EcoRI digests were selected for construction of a fractionated genomic library from which to identify other genes clustered alongside the putative dothistromin genes. Numbers shown indicate sizes of hybridizing bands in kb.

Protoplasts of *D. septosporum* dothistromin-deficient mutants FJT1 (∆*dotA*) and FJT12 (∆*vbsA*) were prepared and transformed using methods described previously [21,22]. FJT1 was transformed with pR280 (*Pa-dotA* construct) and FJT12 with pR283 (*Pa-vbsA* construct); in both cases circular plasmids were co-transformed with plasmid pBC-phleo (pR224, [23]) to enable selection on DM medium containing 7 µg/mL phleomycin (Apollo Scientific Ltd., Stockport, UK). After single spore purification, putative complemented transformants were analyzed for the presence of *Pa-dotA* or *Pa-vbsA* genes by PCR as shown in Supplementary Figure S1. To assay dothistromin production, *D. septosporum* mutants complemented with the *Pa-dotA* or *Pa-vbsA* gene were grown in Dothistroma broth [8] as described above for *P. arachidicola*. After 11 days mycelia were harvested by filtration and the growth media analyzed for dothistromin content with TLC as described previously [22].

### 2.7. Bioinformatics

Sequence data were assembled into contigs using VectorNTI (Invitrogen) and analyzed using VectorNTI or MacVector (Accelrys Inc., San Diego, CA). Sequence comparisons were performed at NCBI (http://www.ncbi.nlm.nih.gov/) using BLAST [24]. GenBank accession numbers for the *P. arachidicola* NZ46 dothistromin gene cluster sequences are GU566729, GU566730 and GU566731.

## 3. Results and Discussion

### 3.1. Isolation of *P. arachidicola* Dothistromin Genes *cypA*, *dotA*, *pksA* and *vbsA* and Functional Identification by Complementation

PCR with degenerate primers targeted to *cypA*, *dotA*, *pksA* and *vbsA* genes yielded products of the expected size (Supplementary Table S1) and with predicted amino acid sequence identities of at least 90% compared to the respective *D. septosporum* homologs. Southern blot analysis confirmed that each sequence was unique and present as a single copy in the *P. arachidicola* genome (Supplementary Figure S1). These PCR products were therefore probably derived from *P. arachidicola* dothistromin biosynthetic genes that have been designated *dotA* (ketoreductase), *cypA* (averufin monooxygenase) *pksA* (polyketide synthase) and *vbsA* (versicolorin B synthase). To clearly distinguish between genes from *P. arachidicola* and *D. septosporum*, the gene names are prefixed by *Pa-* or *Ds-*, respectively, in this article.

Full sequences for *Pa-cypA*, *Pa-dotA*, *Pa-vbsA* genes and a partial sequence for *Pa-pksA* were obtained from library clones. For each of these, nucleotide and amino acid identities were much higher when compared to *D. septosporum* homologs than to *A. parasiticus* (AF cluster) or *A. nidulans* (ST cluster) homologs, as would be expected for vertical inheritance (Table 1; genes in bold type).

**Figure 1:**
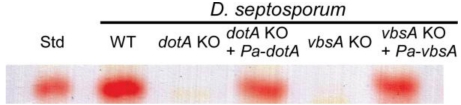
Thin layer chromatography (TLC) assay of dothistromin production by *D. septosporum* WT (NZE10), *dotA* and *vbsA* knockouts (KO) and these knockouts transformed with *P. arachidicola Pa-dotA* and *Pa-vbsA* genes. The red bands corresponding to the dothistromin standard (Std) show that the *P. arachidicola Pa-dotA* and *Pa-vbsA* genes complemented the *D. septosporum* mutations.

**Figure S2:**
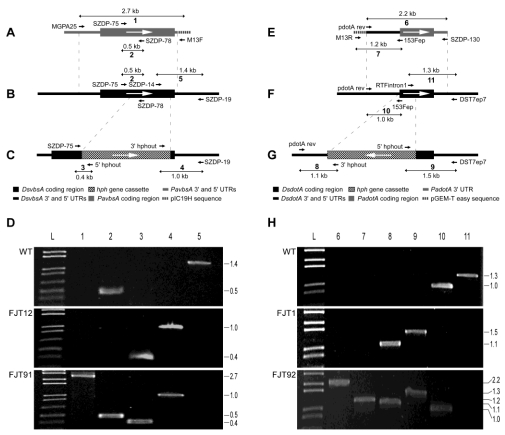
PCR identification of complemented *D. septosporum vbsA* and *dotA* gene knockout mutants with *P. arachidicola* genes. Schematic illustrations are shown of genomic regions for (**A** and **E**) *Pa-vbsA* (A) and *Pa-dotA* (E) used for complementation, (**B** and **F**) *Ds-vbsA* (B) and *Ds-dotA* (F) in wild-type *D. septosporum* (WT), with dotted lines (A to B, E to F) indicating homologous regions in the two species, (**C** and **G**) disrupted *Ds-vbsA* (C) and *Ds-dotA* (G) in mutant *D. septosporum* strains FJT12 (*∆Ds-vbsA*) and FJT1 (*∆Ds-dotA*) used as transformation hosts for complementation, with dotted lines (B to C, F to G) indicating regions replaced with the hygromycin resistant cassette in *D. septosporum*. In A-G, primer binding sites and expected product sizes (kb) are indicated. (**D** and **H**) PCR products amplified from *D. septosporum* WT, *∆Ds-vbsA* mutant FJT12 and *∆Ds-dotA* mutant FJT1, and from the respective mutants complemented with *P. arachidicola Pa-vbsA* (FJT91, D) or * Pa-dotA* (FJT92, H). L: 1 kb plus ladder. PCR products in lanes 1-11 correspond to PCR reactions numbered in A-G. PCR product sizes are in kb.

Functional analysis was performed to confirm that *P. arachidicola* genes are involved in dothistromin biosynthesis. No transformation system has been developed for *P. arachidicola* and attempts to make gene replacement mutants in this species were not successful (results not shown). Instead, two of the *P. arachidicola* genes were analyzed by functional complementation of previously characterized dothistromin-deficient *D. septosporum* gene-replacement mutants [10,21]. Transformation of *Pa-dotA* into a *Ds-dotA* mutant and of *Pa-vbsA* into a *Ds-vbsA* mutant was confirmed by PCR (Supplementary Figure S2). PCR products of *Pa-vbsA* and *Pa-dotA* genes integrated into the *D. septosporum* transformants were sequenced to ensure that they matched *P. arachidicola* and not *D. septosporum* dothistromin genes. Transformation of either *Pa-dotA* or *Pa-vbsA* in the respective *D. septosporum* mutants restored the ability to produce dothistromin (Figure 1), confirming that the *Pa-dotA* and *Pa-vbsA* gene products are functional in dothistromin biosynthesis. Complementation was not done for *Pa-cypA* as no corresponding *D. septosporum* mutant was available, or for *Pa-pksA* because only partial gene sequence was obtained during this study.

### 3.2. Regulation of Dothistromin Biosynthesis in P. Arachidicola

**Figure 2:**
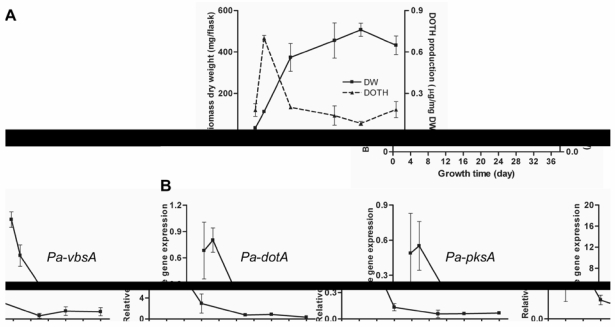
(a) Dothistromin (DOTH) is produced by *P. arachidicola* during early exponential phase in culture. Values are means and standard deviations of three biological replicates, grown in Dothistroma broth (DB) liquid medium. (b) *P. arachidicola* dothistromin biosynthetic genes are most highly expressed during early stages of growth in culture. Semi-quantitative RT-PCR of the three biological replicates in (a) showed the same gene expression patterns and the results from one of these are shown in (b). Values shown are means and standard deviations of three technical replicates. The Y axis shows expression levels of dothistromin genes relative to expression of the β-tubulin gene in *P. arachidicola*.

The production of dothistromin by *P. arachidicola* and expression of dothistromin genes were assessed over a time-course in culture (dothistroma broth; DB). Figure 2 shows that both the rate of dothistromin biosynthesis (µg/mg DW biomass) and expression of *Pa-dotA*, *Pa-vbsA* and *Pa-pksA* genes were highest during early exponential growth phase. These results are very similar to those reported for *D. septosporum* [8], where an unusual early growth-stage expression of dothistromin biosynthesis was shown. The experiment was repeated with potato dextrose broth instead of DB and similar results (not shown) obtained to those found in *D. septosporum* [8]. Although further studies under different growth conditions need to be assessed, * P. arachidicola* and *D. septosporum* do appear to show a different pattern of regulation for dothistromin biosynthesis compared to that for aflatoxin biosynthesis by *Aspergillus* spp., which predominantly involves late-exponential/stationary phase production [9]. 

The discovery that *P. arachidicola*, like *D. septosporum*, produces dothistromin during early exponential growth stage in culture suggests that the pattern of regulation may have some functional importance for the role of dothistromin. The timing of dothistromin biosynthesis *in planta* is not known for *D. septosporum*, but the appearance of red bands in pine needles is secondary to the appearance of necrotic lesions. This, and other reasoning, led us to propose that dothistromin is mainly produced during a period of rapid growth by *D. septosporum* that occurs following nutrient release from dead pine tissue, and that the role of dothistromin may be to inhibit the growth of other needle-dwelling competitor fungi [6]. Although many aspects of this hypothesis remain to be investigated, the toxicity of dothistromin to some needle-dwelling fungi has been demonstrated [6]. Whether dothistromin has any biological role in the *P. arachidicola*-peanut interaction is even less well known. It is not known if dothistromin is produced in peanut leaves infected with *P. arachidicola*, although *P. arachidicola* does have a latent phase *in planta* prior to its necrotrophic phase [17].

### 3.3. Identification and Characterization of Three Groups of Dothistromin Genes

**Figure 3:**
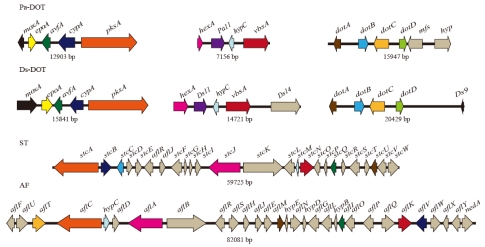
Comparison of dothistromin gene arrangements in *P. arachidicola* (Pa-DOT) and *D. septosporum* (Ds-DOT) with sterigmatocystin (ST) genes in *A. nidulans* and aflatoxin (AF) genes in *A. parasiticus*. Arrows represent genes and show direction of transcription. Black lines connecting genes show physical linkage and sizes of these regions are shown below (not drawn to scale). Putative orthologs from the different species are shown in matching colors.

*P. arachidicola* genomic library clones that hybridized in Southern blots to *Pa-dotA* (14.7 kb clone), *Pa-vbsA* (5.8 kb clone), *Pa-cypA* (6.3 kb clone) and *Pa-pksA* (5.2 kb clone) (Supplementary Figure S1) were sequenced. The *Pa-cypA* and *Pa-pksA* clones were found to be contiguous as they both overlapped with a *Pa-pksA* gene fragment produced by degenerate PCR (Section 3.1). Sequences were extended by inverse PCR as described above to yield the three Pa-DOT contigs shown in Figure 3.

**Figure S3:**
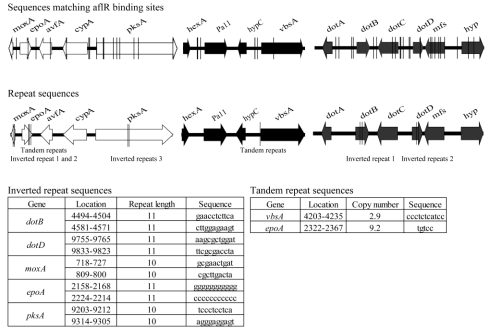
Positions of putative AflR binding sites (top) and repeated sequences (bottom) are shown as vertical lines in the three mini-cluster groups of *Passalora arachidicola* dothistromin genes. The tables show details of inverted or tandem repeats shown in the diagram, with locations numbered according to the gene and GenBank sequences GU566729, GU566730 or GU566731. See main text, Table 1 and Figure 3 for information about the dothistromin genes.

Examination of the predicted genes in the *P. arachidicola* clones revealed a remarkable similarity with dothistromin mini-clusters in *D. septosporum*. As shown in Figure 3, the order and orientation of putative dothistromin genes (colored) in the *P. arachidicola* clones and *D. septosporum* mini-clusters are highly conserved, with only two notable differences. The first main distinguishing feature is that the *moxA* gene is in a different orientation with respect to the rest of the grouped genes, being divergently transcribed from *Pa-epoA* in *P. arachidicola* but in the same orientation as *Ds-epoA* in *D. septosporum*. The reason for this distinction is unclear. The intergenic regions between the *epoA* and *moxA* genes are of similar length (529 bp in *D. septosporum* and 485 bp in *P. arachidicola*), show 53% nucleotide identity and have no obvious repeated sequences or unusual GC composition (Supplementary Figure S3). However, the adjacent *Pa-epoA* gene sequence contains evidence of genomic rearrangement, as discussed below, and this may be related to the inversion of *Pa-moxA*. 

A second notable difference in gene synteny between the two species occurs next to the *dotA-dotD* gene mini-cluster region (Figure 3, right). Whilst *Ds-dotD* is flanked by a 10.6 kb region devoid of genes [10], *Pa-dotD* is only 140 bp away from a putative MFS transporter gene (*Pa-mfsA*), which is closely followed by another open reading frame (*hyp*, of unknown function). There is a high level of nucleotide identity (54%) between the 140 bp *Pa-dotD*: *Pa-mfs* intergenic region and the corresponding region next to *Ds-dotD* and no clear difference in GC composition in this region (see Supplementary Figure S3 and [10]). The *Pa-mfsA* gene is unrelated to the *Ds-dotC* MFS transporter gene, with only 13% amino acid identity. 

As well as an overall conservation of dothistromin gene synteny, *D. septosporum* and *P. arachidicola* dothistromin genes are similar in sequence, with nucleotide identities ranging from 66–86% and predicted amino acid identities of 73–96% (Table 1). The most notable exception to this is between the *epoA* genes, with only 30% nt and 22.5% aa identity. The *Ds-epoA* gene has no homolog in the AF or ST clusters but is predicted to encode a putative 420 aa epoxide hydrolase. Whilst its function is unknown, hypotheses of a role in biosynthesis or detoxification have been proposed [22]. In contrast, *Pa-epoA* is a pseudogene with three stop codons in the predicted coding region and replacement of approximately 650 bp of expected *epoA* coding sequence with a 200 bp repeat-rich region. In addition to this, although both *Ds-epoA* and *Pa-epoA* each have one predicted intron, the positions and sizes of these introns differ. Repeat-rich regions have been associated with other fragmented secondary metabolite gene clusters such as an indole-diterpene cluster in the grass endophyte *Neotyphodium lolii* [12].

Like *D. septosporum*, the putative *P. arachidicola* dothistromin genes have sequences matching binding sites for the AF/ST pathway-specific regulatory protein AflR. In both species, all but one of the dothistromin genes have putative AflR-binding sites just upstream of the coding region, or in an intergenic region shared with a divergently transcribed gene (Supplementary Figure S3). The only genes devoid of upstream AflR sites are *Ds-avfA* and *Pa-avfA*. Since *Ds-avfA* is co-regulated along with other dothistromin genes [10] this suggests that additional regulatory mechanisms are in place. A putative *aflR* ortholog has recently been found in the newly completed *D. septosporum* genome sequence but whether it is involved in the regulation of dothistromin biosynthesis is not yet known. 

The high level of sequence similarity between *P. arachidicola* and *D. septosporum* genes is consistent with the very close phylogenetic relatedness of the *Dothistroma* and *Passalora* genera recently suggested by Crous *et al.* [25]. In contrast, *A. nidulans* and *A. parasiticus*, although belonging to different sections of the genus *Aspergillus*, have a very different gene order (Figure 3) and amino acid identities for homologs of the dothistromin genes range from 55–90% [26]. In general, *P. arachidicola* genes showed a higher level of identity to *A. parasiticus* than to *A. nidulans* genes (Table 1), but this trend was not observed with all genes examined.

**Table 1:** Dothistromin (doth) genes and their predicted gene products, with pair-wise comparisons of nucleotide (nt) and amino acid (aa) identities of *P. arachidicola* (Pa) and *D. septosporum* (Ds) shown in the left panel. Amino acid (aa) identities of Pa and Ds gene products to those of ST/AF orthologs from *A. nidulans* (An) and *A. parasiticus* (Ap) are in the middle panel, and predicted intron numbers for all genes on the right. Horizontal lines distinguish the three groups of Pa genes; these correspond to the Ds mini-clusters shown in Figure 3. Genes shown in bold type are those initially identified by degenerate PCR. Only partial gene sequences for *Pa-pksA*, *Pa-moxA* and *Pa*-*hexA* are available, hence intron numbers for these are shown in parentheses.

doth gene	Putative function	% nt ID	% aa ID	ST/AF ortholog		% aa ID				Intron number			
		Pa/Ds	Pa/Ds	An	Ap	Pa/An	Ds/An	Pa/Ap	Ds/Ap	Pa	Ds	An	Ap
***dotA***	ketoreductase	81.8	95.8	*stcU*	*aflM*	78.8	79.1	79.1	80.2	2	2	2	2
*dotB*	oxidase	77.9	84.3	*stcC*	*-*	15.6	24.0	-	-	0	0	0	-
*dotC*	MFS transporter	76.1	82.3	-	*aflT*	-	-	25.7	31.2	2	3	-	5
*dotD*	thioesterase	74.3	75.5	*stcA*	*aflC*	28.1	37.9	31.6	34.8	0	0	2	5
*mfs*	MFS transporter	*-*	*-*	-	*aflT*	-	-	14.3		7	*-*	-	*-*
***pksA***	polyketide synthase	77.9	88.7	*stcA*	*aflC*	52.5	57.0	54.5	54.8	(2)	2	2	5
***cypA***	monooxygenase	82.3	92.8	*stcB*	*aflV*	55.5	59.8	60.0	59.3	2	2	3	2
*avfA*	oxidase	66.4	73.3	*stcO*	*aflI*	47.0	43.7	46.9	47.8	0	1	0	0
*epoA*	epoxide hydrolase	30	22.5	-	-	-	-	-	-	1	1	-	-
*moxA*	monooxygenase	71.9	88.9	*stcW*	*aflW*	52.5	59.0	57.3	55.1	(3)	5	3	0
*hexA*	fatty acid synthase	69.3	73.8	*stcJ*	*aflA*	40.8	41.3	47.0	48.8	(0)	3	1	2
*Pa11*	unknown	81.1	85.2	-	-	-	-	-	-	3	2	-	-
*hypC*	anthrone oxidase	77.1	86.7	*stcM*	*hypC*	46.9	47.9	37.8	35.2	0	0	0	0
***vbsA***	ver. B synthase	85.8	94.8	*stcN*	*aflK*	70.9	69.1	71.2	72.0	1	1	2	1

The conservation of gene order and sequence between *D. septosporum* and *P. arachidicola* is remarkable. It seems that a very similar ‘fragmented cluster’ of dothistromin genes is present in these two species. Whether the fragmented clusters are present in equivalent regions in the two species is not known, although in *D. septosporum* the three mini-clusters are all located on one 1.3 Mb chromosome [22]. The fragmentation may be even more severe than previously supposed [10]: a recent re-evaluation of *D. septosporum dotB*, *dotD* and *epoA* genes sheds doubt on whether they should be classified as ‘dothistromin genes’ [27]. The discovery that *Pa-epoA* is a pseudogene adds support to notion that *Ds-epoA* may not be required for dothistromin biosynthesis. The availability of the genome sequence of *D. septosporum* (sequenced by the Joint Genome Institute) will enable us to examine the full extent of dothistromin gene organization, assess the regulation of these genes and develop new hypotheses about the evolution of secondary metabolite gene clusters. 

## 4. Conclusions

Dothistromin genes were identified in the peanut pathogen *P. arachidicola* and two of them confirmed by heterologous complementation to be involved in dothistromin biosynthesis. One aim of this study was to determine if the early growth-stage production of dothistromin seen in *D. septosporum* is seen in other dothistromin-producing fungi and our results showed that *P. arachidicola* does indeed appear to share this pattern of regulation. The biological role of dothistromin, and whether the expression patterns seen in culture are relevant to any such role, remains to be determined. Another aim was to compare the organization of dothistromin genes in *D. septosporum* and *P. arachidicola*. A remarkable level of gene synteny and sequence conservation was seen, in keeping with the close phylogenetic relatedness of these species and supporting the hypothesis of vertical transmission of these secondary metabolite genes. DNA sequence differences between *D. septosporum* and *P. arachidicola* added evidence to suggest that *Ds-epoA* may not be required for dothistromin biosynthesis. Furthermore, the differences highlighted possible hot-spots for gene reorganization that may be valuable in future efforts to understand the evolution of fungal metabolite gene clusters.

## Appendix

**Table S1:** Primers used in this study.

Target gene	Primer name (in-house number)	Sequence (5’ - 3’)	Size of PCR product (bp)
Primers used for initial amplification of dothistromin genes by degenerate PCR			
*Pa-dotA*	Keto-ver5’ (#356)	GGIATHGGIGCIGCIATHGC	708
	Keto-ver3’ (#355)	TCRTCIACYTGYTCRTCIGTRAA	
*Pa-cypA*	JSdmo1r (#132)	CGTCAGTGATGATGTCCG	949
	dmoCA (#119)	ATTATGGACGCCACTGCTGG	
*Pa-vbsA*	vbs-1aF (#362)	TNTTYGACTGGTAYCARTAYAC	1351
	vbs-4R (#366)	TAGTTBAGDATYTCBTCRTCSGTCTG	
*Pa-pksA*	pksCB2 (#152)	TGAAGAAGTATGTCGCCG	1306
	OTpks KO/2	CTGTCTAGAGTGTTGGTCTCCATCC	
Primers used for dothistromin gene expression by RT-PCR (Figure 2) (* indicates primer flanks an intron to prevent gDNA amplification)			
*Pa-tub*	SZDP-146 (#865)*	CGGCTCCGGCGTGTACAATG	214
	SZDP-147 (#866)	GGCCCAGTTGTTTCCAGCAC	
*Pa-dotA*	SZDP-143 (#862)*	ACAGGTTGCGAAGATGATGG	474
	SZDP-145(#864)*	AGGCCAACACGTTCTAGAGG	
*Pa-pksA*	MGPA65 (#803)	GTCTCCAGAATTGCCTTGAC	665
	SZDP-142 (#861)*	TCACATTATGTGGTTCGGGC	
*Pa-vbsA*	MGPA31 (#768)	GGGCTCGATGCTCAATACTC	366
	SZDP-140 (#859)*	CGATTGTGGAGACCCTCTTG	
Primers used for construction of the complementation construct pR280			
*Pa-dotA* orf and 3’UTR	SZDP-137 (#724)	CACACCACCTCATCTCCCATAATGTCTGCCGACAACTTCC	1276
	SZDP-130 (#599)	GCGGCCGCTCCCTCTTATCGTTTCATAG	
*Ds-dotA* promoter	SZDP-135 (#722)	ATAGGGCGAATTGGGTACCG	962
	SZDP-136 (#723)	GGAAGTTGTCGGCAGACATTATGGGAGATGAGGTGGTGTG	
Primers used for inverse PCR			
*Pa-dotA* end	SZDP-82 (#538)	GCAACGAAGAATTGACCACG	1000
	SZDP-83 (#539)	GATATGTACGCTGCTGTTGC	
*Pa-moxA* end	MGPA66 (#804)	TGAGCCTGTGGCTCCTGTTG	2100
	MGPA69 (#807)	GTACTGTCTGCGCACAACTC	
*Pa-pksA* end	MGPA22 (#757)	GATCTCCCTTCGAGTCTTTG	900
	MGPA35 (#772)	TCGACAGCTTGAGCTCTATG	
*Pa-hexA* and *Pa-vbsA* ends	MGPA46 (#783)	TGGTGCCGTGGTCATCCTAC	2200
	MGPA79 (#886)	ATCGCCGAACACTTCCACTC	
Primers used for identification of gene knockout and complementation (Figure S2)			
**Reaction**	**Primer name**	**Sequence (5’ - 3’)**	**Product (bp)**
1	MGPA25 (#762)	GGGACATGTTGCTGGTTGTG	2691
	M13 forward	GTAAAACGACGGCCAG	
2	SZDP-75 (#531)	CAGTCCACGTTGCGTATCCA	480
	SZDP-78 (#534)	GGCCGAGGATGATGGTGTCT	
3	SZDP-75 (#531)	CAGTCCACGTTGCGTATCCA	370
	5’hphout (#35)	GAATCTCCGGTGTGGAAGA	
4	3’hphout (#36)	TCCTTGAACTCTCAAGCCTACAG	953
	SZDP-19 (#475)	GTCGGATCGAAGCTCTTCAT	
5	SZDP-14 (#470)	GTGTCGGCCAGAACATGCAA	1389
	SZDP-19 (#475)	GTCGGATCGAAGCTCTTCAT	
6	pdotA rev (#679)	GTCTCTTAAGAATCTTCGTCGCTCAGCT	2234
	SZDP-130 (#599)	GCGGCCGCTCCCTCTTATCGTTTCATAG	
7	M13 reverse	CAGGAAACAGCTATGAC	1183
	153Fep (#59)	GTCGACGGACATTATGGGAGATG	
8	pdotA rev (#679)	GTCTCTTAAGAATCTTCGTCGCTCAGCT	1077
	3’hphout (#36)	TCCTTGAACTCTCAAGCCTACAG	
9	5’hphout (#35)	GAATCTCCGGTGTGGAAGA	1516
	DsT7ep7 (#701)	ATTCGGCTACATGCCCTACAC	
10	pdotA rev (#679)	GTCTCTTAAGAATCTTCGTCGCTCAGCT	971
	153Fep (#59)	GTCGACGGACATTATGGGAGATG	
11	RTFintron1 (#73)	CTGGTGATGTACGTTGTAAAC	1315
	DsT7ep7 (#701)	ATTCGGCTACATGCCCTACAC	
